# Energy and phosphorus digestibility of feed ingredients did not differ between Jeju Island native pigs and commercial Landrace by Yorkshire crossbred pigs

**DOI:** 10.5713/ab.260177

**Published:** 2026-05-07

**Authors:** Hyunwoong Jo, Yeojin An, Beob Gyun Kim

**Affiliations:** 1 Department of Animal Science, Konkuk University, Seoul, Korea

**Keywords:** Digestibility, Energy, Feedstuffs, Jeju Island Native Pigs, Phosphorus, Swine

## Abstract

**Objective:**

This study aimed to determine energy and phosphorus (P) digestibility of feed ingredients fed to Jeju Island native pigs (JINP) and Landrace×Yorkshire (LY) pigs.

**Methods:**

In experiment 1, digestible energy (DE) and metabolizable energy (ME) were measured in 5 ingredients using 12 JINP (30.9±3.3 kg) and 12 LY barrows (52.3±2.8 kg). A diet based on corn and soybean meal, along with 5 experimental diets containing each test ingredient, were used. Twelve pigs from each breed were allotted to a replicated 6×5 incomplete Latin square design, with 6 diets and 5 periods. In experiment 2, standardized total tract digestibility (STTD) of P in the 5 ingredients was measured using 12 JINP (66.8±9.3 kg) and 12 LY barrows (42.8±4.4 kg). Five experimental diets were prepared, each utilizing a different test ingredient as the sole P source, in addition to a P-free diet that was also formulated. The design structure was identical to that of experiment 1.

**Results:**

No interactions between breed and ingredient were observed for DE and ME. The DE and ME of the 5 feed ingredients differed significantly (p<0.001) regardless of pig breed, whereas no breed effects were noted in the DE and ME of the test ingredients. The interaction between breed and ingredient was significant (p = 0.014) in ME:DE but not in DE:gross energy (GE) or ME:GE. No interaction was observed between breed and ingredient regarding the apparent total tract digestibility (ATTD) or STTD of P. The breed effect was noted in the ATTD of P in the test ingredients (p<0.001), but not in the STTD of P. The ATTD and STTD of P in the 5 feed ingredients varied independently of breed (p<0.001).

**Conclusion:**

No significant differences were observed in energy and P digestibility between JINP and LY pigs. The ranking of energy and P digestibility among ingredients was similar for both JINP and LY pigs.

## INTRODUCTION

Energy and phosphorus (P) are the most critical nutrients in swine diets, as energy is required for maintaining and optimizing the growth performance of pigs [1] and P is essential for bone formation and metabolism [2]. Inaccurate determination of the digestibility of energy and P in the feed can result in poor performance of pigs and increased feed costs [2,3]. Thus, accurate evaluation of energy and P digestibility in feed ingredients is essential for precise swine diet formulation. The digestibility experiments require housing pigs in metabolism crates for the separate collection of feces and urine [4]. Nutritional values of feed ingredients are often determined using commercial crossbred pigs, such as Landrace× Yorkshire (LY) pigs. However, the rapid growth of LY pigs may lead to limited space allowance, which makes handling them challenging [5].

To overcome these issues, the pigs used in the present experiments were developed from Jeju Island native pigs (JINP) as laboratory animals [6]. The birth weight and weaning weight of the pigs are generally 0.93 and 5.6 kg, respectively, and the mean body weight (BW) is approximately 30.0 and 65.0 kg at 6 and 12 months of age, respectively. The age at first estrus is 5 to 6 months. In addition, the gestation period is 111 to 114 d, with an average litter size of 7.2, piglets weaned per litter of 6.8, and 1.89 farrowings per year. Compared with LY pigs, the pigs used in the present experiments have a smaller mature body size and a slower growth rate [7]. Therefore, the small-size JINP have the potential to be a model for determining energy and P utilization by LY pigs. To the best of our knowledge, however, no studies have been conducted to determine the energy and P digestibility of feed ingredients in JINP. In addition, the relationship of nutrient utilization between JINP and LY pigs has not been documented. Therefore, the objectives of the present study were to determine digestible energy (DE), metabolizable energy (ME), and P digestibility of feed ingredients fed to small-size JINP and LY pigs.

## MATERIALS AND METHODS

The 5 test ingredients were dehulled soybean meal (SBM) produced in Korea, corn gluten feed (CGF) produced in China, copra meal (CM) produced in the Philippines, palm kernel expellers (PKE) produced in Indonesia, and sesame expellers (SEP) produced in Korea. These ingredients were used in both experiments 1 and 2 (Table 1).

### Experiment 1. Energy utilization

#### Animals, diets, and experimental design

Twelve LY barrows with a mean initial BW of 52.3±2.8 kg (15 wk of age) and 12 small-size Jeju Island native barrows (Cronex) with a mean initial BW of 30.9±3.3 kg (20 wk of age) were used. The basal diet mainly contained corn and SBM, and 5 additional diets were formulated containing 25% of SBM, CGF, CM, PKE, or SEP (Table 2). All diets contained the same proportion of corn:SBM of 4.11:1. A vitamin-mineral premix was included in the diets to meet or exceed the nutrient requirement estimates [2]. The 12 crossbred pigs were individually housed in metabolic cages and assigned to a replicated 6×5 incomplete Latin square design with 6 diets and 5 periods using a spreadsheet-based program to balance potential residual effects [8]. The same experimental design was employed for the 12 JINP.

#### Feeding and sample collection

Daily feed allowance was calculated as 2.7 times the estimated maintenance requirement for energy (i.e., 197 kcal ME/kg BW^0.60^; NRC [2]) based on the BW of pigs measured at the end of each period and divided into 2 equal meals at 08:00 and 16:30 h. Each period consisted of 5 d of adaptation period and 5 d of collection period. Feces were collected following the marker-to-marker procedure [9] using chromic oxide as an indigestible marker. On days 6 and 11 of each experimental period, chromic oxide (0.5% of the diet) was added to the morning meal for initiation and termination of the fecal collection, respectively. Fecal collection was initiated at the first appearance of the marker color in the feces and terminated at the second appearance of the marker. From day 6 at 14:00 h to day 11 at 14:00 h, urine was continuously collected in a bucket containing 200 mL/d of 6 *N* HCl [10]. At the end of each period, a subsample of urine was filtered through a cotton sheet and collected in a 200-mL bottle. The collected fecal and urine samples were immediately stored at −20°C for further chemical analyses.

#### Chemical analyses

The frozen fecal samples were dried in a forced-air drying oven until they reached a constant weight and were finely ground before chemical analyses. The ingredients, diets, and fecal samples were analyzed for dry matter (DM; method 930.15), crude protein (CP; method 990.03), ash (method 942.05), calcium (Ca; method 927.02), and P (method 964.06) using the procedures described in the AOAC [11]. The ingredients and diets were analyzed for ether extract (method 920.39), amylase-treated neutral detergent fiber (method 2002.04), and acid detergent fiber (method 973.18). Thawed urine samples were placed on cotton balls and lyophilized for 24 h. The ingredients, diets, ground fecal samples, and lyophilized urine samples were analyzed for gross energy (GE) using a bomb calorimeter (Parr 6400 bomb calorimeter; Parr Instruments).

#### Calculations and statistical analyses

The DE and ME concentrations of the ingredients were calculated using a difference procedure [4]. Briefly, the DE and ME of the experimental diets were calculated by subtracting fecal energy and both fecal and urinary energy from energy intake, respectively. The energy concentration in the basal diet was divided by 0.971 to obtain the energy concentrations of corn and SBM. The contribution of DE and ME from the basal diet to the test diets was then subtracted from the DE and ME of the test diets to calculate the contributions of DE and ME from the test ingredients in the test diets. These values were then divided by the inclusion rate (0.25) of the test ingredient to calculate the DE and ME of each test ingredient.

Data were analyzed using the MIXED procedure in SAS (SAS Institute). The statistical model included breed, ingredients, and breed×ingredients interactions as fixed variables, and replication, animal within replication, and period within replication as random variables. The PDIFF option with Tukey’s adjustment was used within breed to separate the least squares means. The experimental unit was the pig and statistical significance was declared at an alpha level of 0.05.

### Experiment 2. Phosphorus digestibility

#### Animals, diets, and experimental design

Twelve LY barrows with a mean initial BW of 42.8±4.4 kg (13 wk of age) and 12 small-size Jeju Island native barrows (Cronex) with a mean initial BW of 66.8±9.3 kg (33 wk of age) were used. Five experimental diets were prepared to contain 30% of each test ingredient as the sole source of P (Table 3). Additionally, a P-free diet was formulated to estimate the basal endogenous losses (BEL) of P. A vitamin-mineral premix was included in all diets to meet or exceed the nutrient requirement estimates [2]. The experimental design and treatment arrangements were same as in experiment 1.

#### Feeding and sample collection

Daily feed allowance was calculated as 2.7 times the estimated maintenance requirement for energy (i.e., 197 kcal ME/kg BW^0.60^; NRC [2]) based on the BW of pigs measured at the beginning of each period and divided into 2 equal meals at 08:00 and 16:30 h. Water was freely accessible throughout the experiment. Each period consisted of 4 d of adaptation period and 4 d of fecal collection period. Feces were collected following the marker-to-marker procedure [9] using chromic oxide as an indigestible marker. The collected feces were immediately stored at −20°C for further chemical analyses.

#### Chemical analyses

The frozen fecal samples were dried in a forced-air drying oven until they reached a constant weight and were finely ground before chemical analyses. The experimental diets were analyzed for DM (method 930.15), CP (method 990.03), ether extract (method 920.39), ash (method 942.05), Ca (method 927.02), P (method 964.06), amylase-treated neutral detergent fiber (method 2002.04), and acid detergent fiber (method 973.18) using the procedures described in the AOAC [11]. Fecal samples were analyzed for P (method 964.06).

#### Calculations and statistical analyses

The apparent total tract digestibility (ATTD) and standardized total tract digestibility (STTD) of P in each experimental diet were calculated using the procedure described by [12]. Briefly, the ATTD of P was calculated based on the total P intake (g) and excretion (g) during the collection period. The BEL of P (mg/kg of DM intake) were calculated for pigs fed the P-free diet. The ATTD of P was corrected by the BEL of P for the calculation of STTD of P.

Outliers were identified as values that deviated more than 1.5 times the interquartile range from the 1st or 3rd quartiles. Data were analyzed using the MIXED procedure in SAS (SAS Institute). The statistical model and pairwise comparison procedures were same as in experiment 1. The experimental unit was the pig and statistical significance was declared at an alpha level of 0.05.

## RESULTS

### Composition of ingredients

On an as-is basis, GE in the 5 ingredients ranged from 3,693 to 5,409 kcal/kg and CP contents ranged from 8.01% to 47.9% (Table 1). The ether extract contents ranged from 1.05% to 18.2%. Ash concentrations ranged from 1.38% to 8.96%. The Ca contents ranged from 0.03% to 1.07%. The P contents ranged from 0.25% to 0.89%. The neutral and acid detergent fiber contents ranged from 6.23% to 65.5% and 2.10% to 46.8%, respectively.

### Experiment 1. Energy utilization

In JINP, energy digestibility of the basal and SBM diets was greater than that of the other experimental diets (p<0.05; Table 4). The DE of the basal and SBM diets was also greater than that of other diets (p<0.05). The ME of the SBM diet was greater than that of the CGF, CM, PKE, and SEP diets (p<0.05) but not different from the basal diet in JINP. In LY pigs, energy digestibility, DE and ME of the basal and SBM diets were greater than those of other experimental diets (p<0.05; Table 5).

No interactions between breed and ingredient were observed for DE and ME (Table 6). The DE and ME of the 5 feed ingredients differed regardless of breed (p<0.001), whereas the breed effects were not observed in DE and ME of the test ingredients. The interaction between breed and ingredient was observed in ME:DE (p = 0.014) but not in DE:GE or ME:GE. The energy utilization of the 5 feed ingredients differed regardless of breed (p<0.001).

### Experiment 2. Phosphorus digestibility

In both JINP and LY pigs, P intake and fecal P output were greater for SEP compared with the other ingredients (p<0.05; Table 7). No interaction was observed between breed and ingredient for the ATTD or STTD of P. The breed effect was observed in the ATTD of P in the test ingredients (p<0.001), but not in the STTD of P. The ATTD and STTD of P in the 5 feed ingredients differed between the breeds (p<0.001).

## DISCUSSION

The pigs used in the present experiments are recognized as suitable laboratory animals because of their slow growth rate and small adult size [7]. However, differences in nutrient digestibility potentially exist between JINP and LY pigs if the 2 breeds differ in digestive function. However, nutrient digestibility has not been compared between JINP and LY pigs. The test ingredients used in the present experiment covered wide ranges of GE, mineral, and fiber contents, which are desirable for examining breed-dependent differences in energy and P digestibility.

The nutrient compositions of the test ingredients in the present work were within the range of previously reported values, except for SEP [2,13–16]. The GE content in SEP was greater than previous values, likely due to the greater ether extract contents and less ash content in the SEP of the present work [2,13,14]. The nutritional deviations in SEP among the studies may have resulted from variations in the intensity and duration of the heat treatment during oil extraction [17].

The DE and ME of all test ingredients in LY pigs obtained in this study were in good agreement with previous values [2,13–16]. The rankings of DE and ME values of the test ingredients in JINP were similar to those observed in LY pigs. The greatest DE and ME values observed in SBM are likely attributed to the greater DE:GE in both breeds among test ingredients. The greater digestibility is likely due to the less fiber content in the SBM compared with the other test ingredients. Dietary fiber interferes with the action of digestive enzymes on substrates in the gastrointestinal tract and increases the passage rate of digesta [18–20]. Despite the lowest DE:GE and ME:GE observed in the pigs fed the SEP diet, the DE and ME values of the SEP were not the lowest among the test ingredients, most likely due to the high GE concentration in the SEP.

In the present experiment, BW differed between the JINP and LY pigs. As pigs grow, their hindgut capacity increases, which allows heavier or older pigs to produce more energy from high-fiber ingredients than lighter or younger pigs [21]. In the present study, however, no difference in energy digestibility was observed between the two breeds, which is likely attributed to the similar fiber digestibility between JINP and LY pigs [22]. Although energy digestibility may increase with BW during the growing phase (e.g., 25 vs. 90 kg), the magnitude of the change is small [23]. This finding agrees with the results of Park and Kim [22], where no difference in energy digestibility was observed between growing and finishing pigs fed a high-fiber diet, but a difference was observed between nursery pigs and growing pigs. These factors explain the similar rankings of DE and ME in the feed ingredients between the two breeds, without any interaction effect.

The ATTD and STTD of P in SBM, CGF, and CM in LY pigs were within the ranges reported in previous studies [2,12,24,25]. However, the ATTD and STTD in SEP were less than those reported in a previous study [2], whereas the ATTD and STTD in PKE were greater than those previously reported [2,26]. These differences in P digestibility among the oil byproducts can be explained by differences in the contents of phytate P, which is not available to pigs [26,27]. The content of phytate P is dependent on temperature during oil extraction [28]. The heat used during the oil extraction process for PKE may have affected the breakdown of phytate P. The lowest STTD of P observed in SEP in both breeds may be attributed to the higher dietary Ca contents, which can negatively affect P digestibility. Excessive Ca intake can disrupt P absorption by forming Ca–phosphate complexes that are less readily digested and absorbed in the gastrointestinal tract [27,29]. These findings are supported by Lee and Stein [30], who reported that the apparent ileal digestibility of P decreased with increasing dietary Ca. In the present work, the SEP diet had a greater Ca content than the other test diets, possibly contributing to the lowest STTD of P observed in SEP. Although the Ca:P in the PKE diet was also not optimal, the STTD of P remained relatively high, which may be attributed to the relatively low contents of phytate P in PKE.

The BEL of P observed for LY pigs in the present study was comparable to previously reported values [31,32]. To our knowledge, however, BEL of P has not been previously reported for JINP. The reason for the greater BEL of P in JINP than in LY pigs observed in the present work remains unclear. Basal endogenous losses of P are affected by several factors, including dietary fiber type and content, feed intake, and ingredient composition [32]; however, these factors were controlled in the present experiment. The BEL of P include P originating from digestive fluid and sloughed mucosal cells [32,33]. Breed-related differences in gastrointestinal microbial profiles may have contributed to the variation in BEL of P between JINP and LY pigs [32,33]. The greater BEL of P in JINP than in LY pigs are potentially due to differences in age and BW [34,35]. The JINP used in this study had an initial age of 33 weeks, whereas the LY pigs were 13 weeks old, as similar BW pigs could not be used due to differences in growth rate between JINP and LY pigs. However, it should be noted that the BEL of P was expressed as milligrams per kilogram DM intake, and thus, feed intake differences due to the BW are readily reflected in the unit for the BEL of P. The greater BEL of P in JINP likely resulted in less ATTD of P compared with LY pigs. However, no difference was observed in the STTD of P between JINP and LY pigs as STTD values accounted for differences in BEL of P in the calculation of STTD of P.

In the present study, no differences in energy and P digestibility were observed between JINP and LY pigs. Previous studies have suggested that genotype-related differences in digestive morphology and gastrointestinal tract length may influence nutrient digestibility [23,36]. In the present study, the digestive tract characteristics of JINP and LY pigs may not differ sufficiently to induce differences in energy and P digestibility. Although differences in metabolic age between JINP and LY pigs may have existed due to differences in growth rate, these differences were not reflected in energy digestibility. Due to limitations in animal availability, it was not possible to use pigs with similar BW. Therefore, further studies are needed to evaluate the gastrointestinal characteristics of JINP and LY pigs.

## CONCLUSION

No difference was observed in the energy and P digestibility between JINP and LY pigs. Furthermore, the ranking of energy and P digestibility among ingredients was similar for both JINP and LY pigs. Therefore, JINP have the potential to be a small-sized model for determining energy and P utilization in LY pigs.

## Figures and Tables

**Table 1:** Analyzed energy and nutrient composition of feed ingredients (as-is basis)

Item	Corn	SBM	CGF	CM	PKE	SEP
Dry matter (%)	87.0	89.4	89.6	89.1	90.1	94.3
Gross energy (kcal/kg)	3,693	4,211	4,118	3,861	4,395	5,409
Crude protein (%)	8.0	47.9	19.0	21.4	15.1	41.2
Ether extract (%)	3.0	1.1	2.1	1.8	5.7	18.2
Ash (%)	1.4	5.9	5.8	7.2	4.6	9.0
Calcium (%)	0.03	0.26	0.11	0.23	0.48	1.07
Phosphorus (%)	0.25	0.43	0.54	0.49	0.45	0.89
Neutral detergent fiber (%)	8.1	6.2	32.8	55.5	65.5	35.7
Acid detergent fiber (%)	2.1	3.2	9.1	31.5	46.8	19.5

SBM, soybean meal; CGF, corn gluten feed; CM, copra meal; PKE, palm kernel expellers; SEP, sesame expellers.

**Table 2:** Ingredients and analyzed nutrient composition of experimental diets in experiment 1 (as-fed basis)

Item	Basal	SBM	CGF	CM	PKE	SEP
Ingredient (%)						
Ground corn	78.10	58.39	58.03	58.07	58.19	59.08
SBM (48% crude protein)	19.00	14.21	14.12	14.13	14.16	14.37
SBM (48% crude protein)	-	25.00	-	-	-	-
CGF	-	-	25.00	-	-	-
CM	-	-	-	25.00	-	-
PKE	-	-		-	25.00	-
SEP	-	-	-	-	-	25.00
Ground limestone	0.80	0.70	1.10	1.00	1.00	0.10
Dicalcium phosphate	1.20	0.80	0.85	0.90	0.75	0.55
Sodium chloride	0.40	0.40	0.40	0.40	0.40	0.40
Vitamin-mineral premix^1)^	0.50	0.50	0.50	0.50	0.50	0.50
Analyzed composition						
Dry matter (%)	87.5	87.8	88.3	88.1	88.7	90.4
Gross energy (kcal/kg)	3,869	3,957	3,929	3,912	4,030	4,319
Crude protein (%)	14.7	22.7	16.8	17.8	17.8	22.7
Ether extract (%)	2.5	2.2	2.4	2.6	4.4	6.6
Ash (%)	4.5	4.9	5.5	5.5	5.1	4.9
Neutral detergent fiber (%)	7.4	7.1	15.8	18.6	22.4	16.0
Acid detergent fiber (%)	2.2	2.5	4.4	9.0	13.0	7.1

1) Provided the following quantities per kilogram of complete diet: vitamin A, 50,000 IU; vitamin D_3_, 10,000 IU; vitamin E, 450 IU; vitamin K, 6.25 mg; thiamin, 8.75 mg; riboflavin, 20 mg; pyridoxine, 12.5 mg; vitamin B_12_, 0.11 mg; pantothenic acid, 62.5 mg; folic acid, 6.25 mg; niacin, 105 mg; biotin, 0.5 mg; Cu, 12.5 mg as copper sulfate; Fe, 125 mg as iron sulfate; I, 1.00 mg as calcium iodate; Mn, 75 mg as manganese sulfate; Se, 0.25 mg as sodium selenite; and Zn, 10 mg as zinc sulfate.

SBM, soybean meal; CGF, corn gluten feed; CM, copra meal; PKE, palm kernel expellers; SEP, sesame expellers.

**Table 3:** Ingredients and analyzed nutrient composition of experimental diets in experiment 2 (as-fed basis)

Item	SBM	CGF	CM	PKE	SEP	P-free
Ingredients (%)						
Cornstarch	44.7	38.5	38.1	37.2	45.1	53.8
SBM (48% crude protein)	30.0	-	-	-	-	-
CGF	-	30.0	-	-	-	-
CM	-	-	30.0	-	-	-
PKE	-	-	-	30.0	-	-
SEP	-	-	-	-	30.0	-
Sucrose	20.0	20.0	20.0	20.0	20.0	20.0
Soybean oil	3.36	2.75	1.82	2.04	1.80	4.00
Gelatin	0.80	7.60	8.90	9.80	2.20	13.3
Cellulose	-	-	-	-	-	4.00
_L_-Lys-HCl (78.8%)	-	-	-	-	-	0.50
_DL_-Met (99.0%)	-	-	-	-	-	0.13
_L_-Thr (98.0%)	-	-	-	-	-	0.29
_L_-Trp (99.0%)	-	-	-	-	-	0.14
_L_-His (98.5%)	-	-	-	-	-	0.21
_L_-Ile (99.4%)	-	-	-	-	-	0.30
_L_-Val (98.5%)	-	-	-	-	-	0.29
Ground limestone	0.26	0.22	0.30	0.11	-	1.63
Potassium carbonate	-	-	-	-	-	0.40
Magnesium oxide	-	-	-	-	-	0.10
Sodium chloride	0.40	0.40	0.40	0.40	0.40	0.40
Vitamin-mineral premix^1)^	0.50	0.50	0.50	0.50	0.50	0.50
Analyzed composition (%)						
Dry matter	95.1	93.8	92.9	93.7	95.2	94.5
Crude protein	14.6	16.6	16.1	16.7	14.5	16.3
Ether extract	1.7	1.5	1.7	2.6	2.9	1.1
Ash	2.7	2.7	3.4	2.3	3.3	3.0
Calcium	0.24	0.16	0.21	0.25	0.36	0.53
Phosphorus	0.17	0.19	0.12	0.12	0.23	0.01
Neutral detergent fiber	2.3	9.9	16.1	18.9	11.7	3.9
Acid detergent fiber	1.0	2.8	9.1	12.8	6.5	3.8

1) Provided the following quantities per kilogram of complete diet: vitamin A, 50,000 IU; vitamin D_3_, 10,000 IU; vitamin E, 450 IU; vitamin K, 6.25 mg; thiamin, 8.75 mg; riboflavin, 20 mg; pyridoxine, 12.5 mg; vitamin B_12_, 0.11 mg; pantothenic acid, 62.5 mg; folic acid, 6.25 mg; niacin, 105 mg; biotin, 0.5 mg; Cu, 12.5 mg as copper sulfate; Fe, 125 mg as iron sulfate; I, 1.00 mg as calcium iodate; Mn, 75 mg as manganese sulfate; Se, 0.25 mg as sodium selenite; and Zn, 10 mg as zinc sulfate.

SBM, soybean meal; CGF, corn gluten feed; CM, copra meal; PKE, palm kernel expellers; SEP, sesame expellers.

**Table 4:** Energy digestibility and metabolizability of Jeju Island native pigs fed experimental diets in experiment 1 (as-fed basis)

Item	Basal	SBM	CGF	CM	PKE	SEP	SEM	p-value
Feed GE (kcal/kg)	3,869	3,957	3,929	3,912	4,030	4,319	-	-
Feed intake (kg/d)	1.31	1.26	1.37	1.36	1.32	1.34	0.11	0.078
GE intake (kcal/d)	5,045^b^	4,939^b^	5,382^ab^	5,319^ab^	5,326^ab^	5,780^a^	440	<0.001
Dry feces output (kg/d)	0.12^c^	0.11^c^	0.23^a^	0.19^b^	0.22^ab^	0.24^a^	0.02	<0.001
GE in dry feces (kcal/kg)	4,046^de^	3,987^e^	4,128^cd^	4,175^c^	4,288^b^	4,515^a^	36	<0.001
Fecal GE output (kcal/d)	496^c^	454^c^	958^a^	790^b^	948^ab^	1,103^a^	68	<0.001
Energy digestibility (%)	90.1^a^	90.8^a^	82.0^c^	85.2^b^	81.9^c^	80.6^c^	0.5	<0.001
DE in diet (kcal/kg)	3,478^b^	3,594^a^	3,221^d^	3,331^c^	3,300^cd^	3,480^b^	21	<0.001
Urine output (kg/d)	2.47	3.04	2.56	2.46	2.52	1.91	0.50	0.062
GE in urine (kcal/kg)	71.6^b^	79.3^b^	73.0^b^	77.0^b^	65.9^b^	124.0^a^	11.1	<0.001
Urinary GE output (kcal/d)	144^c^	204^ab^	158^bc^	183^abc^	148^c^	207^a^	12	0.002
Energy metabolizability (%)	87.1^a^	86.8^a^	79.0^c^	81.7^b^	79.1^c^	77.0^c^	0.6	<0.001
ME in diet (kcal/kg)	3,374^ab^	3,433^a^	3,104^c^	3,197^c^	3,188^c^	3,325^b^	24	<0.001

Each least squares mean represents 10 observations.

The animals were obtained from Cronex.

a–e Within a row means without a common superscript differ (p<0.05).

SBM, soybean meal; CGF, corn gluten feed; CM, copra meal; PKE, palm kernel expellers; SEP, sesame expellers; SEM, standard error of the means; GE, gross energy; DE, digestible energy; ME, metabolizable energy.

**Table 5:** Energy digestibility and metabolizability of Landrace×Yorkshire pigs fed experimental diets in experiment 1 (as-fed basis)

Item	Basal	SBM	CGF	CM	PKE	SEP	SEM	p-value
Feed GE (kcal/kg)	3,869	3,957	3,929	3,912	4,030	4,319	-	-
Feed intake (kg/d)	2.00^ab^	1.92^b^	2.11^a^	2.04^ab^	2.01^ab^	2.05^a^	0.11	<0.001
GE intake (kcal/d)	7,728^cd^	7,603^d^	8,300^b^	7,979^bcd^	8,096^bc^	8,834^a^	423	<0.001
Dry feces output (kg/d)	0.15^d^	0.16^d^	0.32^b^	0.26^c^	0.29^bc^	0.38^a^	0.02	<0.001
GE in dry feces (kcal/kg)	4,015^c^	3,994^c^	4,168^b^	4,162^b^	4,287^b^	4,568^a^	41	<0.001
Fecal GE output (kcal/d)	616^d^	634^d^	1,351^b^	1,093^c^	1,238^bc^	1,737^a^	57	<0.001
Energy digestibility (%)	91.9^a^	91.7^a^	83.6^c^	86.2^b^	84.5^bc^	80.2^d^	0.6	<0.001
DE in diet (kcal/kg)	3,556^a^	3,626^a^	3,284^d^	3,374^c^	3,404^bc^	3,465^b^	23	<0.001
Urine output (kg/d)	4.01	4.77	4.08	3.94	3.70	4.24	0.38	0.144
GE in urine (kcal/kg)	60.8^ab^	53.5^b^	65.3^ab^	63.4^ab^	67.9^ab^	85.2^a^	7.0	0.016
Urinary GE output (kcal/d)	228^b^	244^b^	247^b^	232^b^	228^b^	331^a^	17	<0.001
Energy metabolizability (%)	89.0^a^	88.4^a^	80.5^c^	83.3^b^	81.6^bc^	76.5^d^	0.6	<0.001
ME in diet (kcal/kg)	3,442^a^	3,497^a^	3,163^c^	3,258^b^	3,288^b^	3,303^b^	25	<0.001

Each least squares mean represents 10 observations.

The animals were obtained from Cronex.

a–d Within a row means without a common superscript differ (p<0.05).

SBM, soybean meal; CGF, corn gluten feed; CM, copra meal; PKE, palm kernel expellers; SEP, sesame expellers; SEM, standard error of the means; GE, gross energy; DE, digestible energy; ME, metabolizable energy.

**Table 6:** Comparison between Jeju Island native pigs and Landrace×Yorkshire pigs for energy concentrations in soybean meal (SBM), corn gluten feed (CGF), copra meal (CM), palm kernel expellers (PKE), and sesame expellers (SEP) in experiment 1

Item	Jeju Island native pigs					Landrace×Yorkshire pigs					SEM	p-value		
		
	SBM	CGF	CM	PKE	SEP	SBM	CGF	CM	PKE	SEP		B	I	B×I
As-fed basis (kcal/kg)														
GE	4,211	4,118	3,861	4,395	5,409	4,211	4,118	3,861	4,395	5,409	-	-	-	-
DE	3,939^a^	2,520^d^	2,947^c^	2,810^cd^	3,372^b^	3,878^w^	2,557^y^	2,923^xy^	3,017^x^	3,107^x^	92	0.706	<0.001	0.123
ME	3,629^a^	2,389^c^	2,739^bc^	2,701^c^	3,092^b^	3,704^w^	2,419^y^	2,800^xy^	2,898^x^	2,805^xy^	100	0.802	<0.001	0.128
Dry matter basis (kcal/kg)														
GE	4,770	4,625	4,404	4,827	5,500	4,770	4,625	4,404	4,827	5,500	-	-	-	-
DE	4,461^a^	2,830^c^	3,361^b^	3,087^bc^	3,430^b^	4,393^w^	2,872^y^	3,334^x^	3,314^x^	3,160^xy^	101	0.754	<0.001	0.152
ME	4,110^a^	2,684^c^	3,124^b^	2,967^bc^	3,145^b^	4,195^w^	2,717^y^	3,193^x^	3,183^x^	2,853^xy^	110	0.736	<0.001	0.167
Energy utilization														
DE:GE	0.94^a^	0.61^c^	0.76^b^	0.64^c^	0.62^c^	0.92^w^	0.62^yz^	0.76^x^	0.69^xy^	0.57^z^	0.02	0.835	<0.001	0.213
ME:DE	0.92^b^	0.95^ab^	0.93^ab^	0.96^a^	0.92^b^	0.95^w^	0.94^w^	0.96^w^	0.96^w^	0.90^x^	0.01	0.096	<0.001	0.014
ME:GE	0.86^a^	0.58^c^	0.71^b^	0.61^c^	0.57^c^	0.88^w^	0.59^yz^	0.73^x^	0.66^xy^	0.52^z^	0.02	0.641	<0.001	0.248

Each least squares mean represents 10 observations.

The animals were obtained from Cronex.

a–d,w–z Within a row of a breed means without a common superscript differ (p<0.05).

SEM, standard error of the means; B, breed; I, ingredient; B×I, interaction between breed and ingredient; GE, gross energy; DE, digestible energy; ME, metabolizable energy.

**Table 7:** Comparison between Jeju Island native pigs and Landrace×Yorkshire (LY) pigs for digestibility of phosphorus (P) in soybean meal (SBM), corn gluten feed (CGF), copra meal (CM), palm kernel expellers (PKE), and sesame expellers (SEP) in experiment 2

Item	Jeju Island native pigs					Landrace×Yorkshire pigs					SEM	p-value		
		
	SBM	CGF	CM	PKE	SEP	SBM	CGF	CM	PKE	SEP		B	I	B×I
n	8	6	9	10	6	10	6	6	8	7				
Feed intake (g/d)	1,705^ab^	1,569^ab^	1,698^ab^	1,433^b^	1,841^a^	1,315^w^	967^x^	1,070^x^	883^x^	1,348^w^	103.1	<0.001	<0.001	0.586
P intake (g/d)	2.85^b^	3.02^b^	2.12^c^	1.67^c^	4.17^a^	2.20^x^	1.82^y^	1.32^yz^	1.04^z^	3.03^w^	0.171	<0.001	<0.001	0.112
Fecal output (g/d)	79.8^c^	163^b^	138^b^	150^b^	269^a^	53.8^x^	92.5^x^	78.9^x^	75.1^x^	172^w^	11.7	<0.001	<0.001	0.021
P in feces (%)	2.86^a^	1.45^b^	0.95^c^	0.71^c^	1.44^b^	2.86^w^	1.43^x^	0.74^y^	0.71^y^	1.46^x^	0.081	0.329	<0.001	0.524
P output (g/d)	2.22^b^	2.31^b^	1.33^c^	1.03^c^	3.84^a^	1.51^x^	1.30^x^	0.56^y^	0.53^y^	2.43^w^	0.135	<0.001	<0.001	<0.001
ATTD of P (%)	22.4^bc^	23.1^abc^	34.8^ab^	37.5^a^	8.20^c^	30.7^y^	29.6^xy^	57.2^w^	46.2^wx^	20.3^y^	4.22	<0.001	<0.001	0.356
STTD of P (%)^1)^	41.3^b^	39.4^b^	60.0^a^	64.3^a^	22.2^c^	41.0^x^	38.5^x^	71.3^w^	60.8^w^	27.9^y^	4.24	0.362	<0.001	0.407

The animals were obtained from Cronex.

1) Values for STTD were calculated by correcting ATTD values for basal endogenous losses of P. The basal endogenous losses of P in Jeju Island native pigs and LY pigs were 332±190 mg/kg and 180±79 mg/kg of dry matter intake, respectively.

a–c,w–z Within a row of a breed means without a common superscript differ (p<0.05).

SEM, standard error of the means; B, breed; I, ingredient; B×I, interaction between breed and ingredient; ATTD, apparent total tract digestibility; STTD, standardized total tract digestibility.

## Data Availability

Upon reasonable request, the datasets of this study can be available from the corresponding author.
